# Study protocol for OptimalTTF-2: enhancing Tumor Treating Fields with skull remodeling surgery for first recurrence glioblastoma: a phase 2, multi-center, randomized, prospective, interventional trial

**DOI:** 10.1186/s12885-021-08709-4

**Published:** 2021-09-09

**Authors:** N. Mikic, F. R. Poulsen, K. B. Kristoffersen, R. J. Laursen, T. L. Guldberg, J. Skjøth-Rasmussen, E. T. Wong, S. Møller, R. H. Dahlrot, J. C. H. Sørensen, A. R. Korshøj

**Affiliations:** 1 Department of Neurosurgery, Aarhus University Hospital, Palle Juul-Jensens Blvd 165, 8200 Aarhus, Denmark; 2 Department of Clinical Medicine, Aarhus University, Palle Juul-Jensens Blvd. 82, 8200 Aarhus, Denmark; 3 Department of Neurosurgery, Odense University Hospital, Kløvervænget 47, 5000 Odense, Denmark; 4 Clinical Institute BRIDGE (Brain Research InterDisciplinary Guided Excellence), University of Southern Denmark, Winsløwparken 19, 5000 Odense, Denmark; 5 Department of Oncology, Aarhus University Hospital, Palle Juul-Jensens Blvd. 99, 8200 Aarhus, Denmark; 6 Department of Neurosurgery, Aalborg University Hospital, Hobrovej 18-22, 9000 Aalborg, Denmark; 7 Department of Oncology, Aalborg University Hospital, Hobrovej 18-22, 9000 Aalborg, Denmark; 8 Department of Neurosurgery, Rigshospitalet, Inge Lehmanns Vej 6, 2100 København Ø, Denmark; 9 Beth Israel Deaconess Medical Center, 330 Brookline Ave, Boston, MA 02215 USA; 10 Harvard Medical School, 25 Shattuck St, Boston, MA 02115 USA; 11 Department of Oncology, Rigshospitalet, Blegdamsvej 9, 2100 København Ø, Denmark; 12 Department of Oncology, Odense University Hospital, Kløvervænget 19, 5000 Odense, Denmark

**Keywords:** Craniectomy | glioblastoma | neuro-oncology | neurosurgery | tumour treating fields |

## Abstract

**Background:**

OptimalTTF-2 is a randomized, comparative, multi-center, investigator-initiated, interventional study aiming to test skull remodeling surgery in combination with Tumor Treating Fields therapy (TTFields) and best physicians choice medical oncological therapy for first recurrence in glioblastoma patients. OptimalTTF-2 is a phase 2 trial initiated in November 2020. Skull remodeling surgery consists of five burrholes, each 15 mm in diameter, directly over the tumor resection cavity. Preclinical research indicates that this procedure enhances the effect of Tumor Treating Fields considerably. We recently concluded a phase 1 safety/feasibility trial that indicated improved overall survival and no additional toxicity. This phase 2 trial aims to validate the efficacy of the proposed intervention.

**Methods:**

The trial is designed as a comparative, 1:1 randomized, minimax two-stage phase 2 with an expected 70 patients to a maximum sample size of 84 patients. After 12-months follow-up of the first 52 patients, an interim futility analysis will be performed. The two trial arms will consist of either a) TTFields therapy combined with best physicians choice oncological treatment (control arm) or b) skull remodeling surgery, TTFields therapy and best practice oncology (interventional arm). Major eligibility criteria include age ≥ 18 years, 1st recurrence of supratentorial glioblastoma, Karnofsky performance score ≥ 70, focal tumor, and lack of significant co-morbidity. Study design aims to detect a 20% increase in overall survival after 12 months (OS12), assuming OS12 = 40% in the control group and OS12 = 60% in the intervention group. Secondary endpoints include hazard rate ratio of overall survival and progression-free survival, objective tumor response rate, quality of life, KPS, steroid dose, and toxicity. Toxicity, objective tumor response rate, and QoL will be assessed every 3rd month. Endpoint data will be collected at the end of the trial, including the occurrence of suspected unexpected serious adverse reactions (SUSARs), unacceptable serious adverse events (SAEs), withdrawal of consent, or loss-to-follow-up.

**Discussion:**

New treatment modalities are highly needed for first recurrence glioblastoma. Our proposed treatment modality of skull remodeling surgery, Tumor Treating Fields, and best practice medical oncological therapy may increase overall survival significantly.

**Trial registration:**

ClinicalTrials.gov Identifier: NCT0422399, registered 13. January 2020.

## Background

Glioblastoma (GBM) is the most common primary brain cancer, while simultaneously being the most devastating in terms of overall survival (OS) and reduction of quality of life. Despite maximum safe surgical resection, radiotherapy with concomitant and adjuvant temozolomide the reported OS is in the range of 12–18 months [1, 2]. GBM has a notoriously high recurrence rate, and the OS after first recurrence is approximately 6–10.4 months, median progression-free survival (PFS) 1.5–2.8 months, and overall, 12-month survival (OS12) 20–34,1% [3] [4, 5]. Once recurrence is observed, the treatment options are limited and there is no established and effective standard of care. Novel treatments are therefore highly warranted.

Tumor Treating Fields (TTFields) is an antimitotic cancer treatment that works with low-intensity (1–3 V/cm) and intermediate frequency (∼200 kHz) electric fields. TTFields is delivered noninvasively through two orthogonal pairs of transducer arrays placed on the shaved scalp. TTFields frequency is tuned for distinct cancer cell lines; as a result, tumor cells are disrupted during mitosis, while healthy quiescent cells are not negatively affected [6].

When TTFields are added to maintenance temozolomide for newly diagnosed GBM, median OS is significantly increased to 20.9 months (HR = 0.63; 95% CI, 0.53–0.76; *P* < 0.001) and PFS to 6.7 months (HR = 0.63; 95% CI, 0.52–0.76; *P <* 0.001) [7]. In the recurrent GBM setting, a randomized trial (EF-11) showed comparable survival outcomes between TTFields monotherapy (median OS, 6.6 months) and best physician’s choice chemotherapy (BPC) (median OS 6.0 months (*p* = 0.27)). Further, the TTFields therapy group had significantly less serious adverse events and better quality of life [5]. Following the EF-11 trial, a registry study analyzed all recurrence GBM patients in the U. S treated with TTFields. (regardless of number of recurrences, KPS, previous treatment) and showed an OS of 9.6 months and OS12 at 44% for patients treated with TTFields monotherapy [8]. Currently TTFields is approved for newly diagnosed GBM and recurrent GBM.

A novel method of TTFields delivery was proposed by Korshøj et al., involving “skull remodelling surgery” (SR-surgery), i.e., craniectomy or burrholes, to create a low resistance pathway for electrical field strength during TTFields therapy. By using finite element analysis to calculate the electric field distribution in a realistic head model [9–11], preclinical research shows that removal of a standard craniotomy bone flap increases the electrical field strength by 60–70% in superficially located tumor sites and expected percentage (30–50%) of tissue in growth arrest [12]. In addition, multiple smaller burr holes are more efficient than single craniectomies for creating increased electrical field strength of equivalent area [12].

These findings [9–12] resulted in a clinical (phase 1) single-arm pilot study. OptimalTTF-1 (NCT02893137) testing the safety and feasibility of combining “skull remodeling surgery” (SR-surgery) with TTFields therapy and BPC chemotherapy for first recurrence glioblastoma (*n* = 15). The trial concluded that the combination of SR-surgery and TTFields therapy was safe and feasible without additional toxicity and with OS at 15.5 months, OS12 at 55%, and PFS at 4.6 months [13].

The current proposed phase 2 trial aims to validate this hypothesis in a randomized comparative setting. This manuscript is based on protocol version 2.0 dated 7th November 2020.

## Methods/design

The study is designed as an investigator-initiated, prospective, multi-center, multi-national, 1:1 randomized, minimax two-stage, comparative, phase 2 trial, investigating efficacy of the intervention [14]. Patients randomized to the control arm will receive TTFields therapy plus physician’s best choice medical oncological therapy (PBC). Patients in the interventional arm will receive SR-surgery in addition to TTFields therapy and PBC treatment. The trial will enroll an expected sample size of 70 patients from 4 sites with Aarhus University Hospital, Denmark, as the Sponsor and coordinating site. The 3 other sites in Denmark are Odense University Hospital, Aalborg University Hospital and Rigshospitalet. The primary outcome will be overall survival at 12-months (OS12). The trial is designed to detect a 20% increase in OS12 in the interventional arm compared to control (from 40% in the control arm to 60% in the interventional arm). Secondary outcomes will include PFS, quality of life (QoL), Karnofsky Performance Score (KPS), and objective response rate (ORR). Interim futility analysis will be conducted after 12 months follow-up of the first 52 patients. The trial will be stopped at interim analysis if the OS12 is equal or higher in the control arm compared to the interventional arm. The trial began in November 2020 and is expected to end in December 2023. The inclusion period is expected to be the first 24 months of the trial.

### Primary endpoint

1. Overall survival rate at 12 months (OS12)

### Secondary endpoints

1. Median PFS.

2. Median OS.

3. OS rate at 24 months and 36 months.

4. PFS rate at 6 months.

5. Overall objective response rate assessed by the modified RANO criteria (ORR).

6. Quality of life assessment (EORTC QLQ-C30 and QLQ-BN20).

7. Cumulative corticosteroid dosage.

8. KPS decline.

9. Adverse events severity and frequency (CTCAE version 5.0).

Survival estimates will be measured from the time of inclusion.

### Trial overview

#### Screening and enrollment

Seventy patients with first recurrence GBM according to RANO [15] criteria will be enrolled. Potential trial participants will be identified at an institutional multidisciplinary neuro-oncological tumor board and subsequently referred for enrollment and eligibility screening. All inclusion scans are assessed by a trained neuroradiologist. Immediately ineligible patients, e.g., due to poor performance status, multifocal disease, significant comorbidity, evidence of extracranial primary tumor, or other excluding circumstances, will not be referred for screening.

### Eligibility criteria

#### Inclusion criteria

- age 18 years or older
- first recurrence GBM based on the RANO criteria
- whole-brain MRI according to the consensus recommendations for a standardized brain tumor imaging protocol in clinical trials not older than 4 weeks from assessment
- estimated survival ≥3 months
- supratentorial tumor location
- focal disease in the vicinity of the previously known tumor or resection cavity,
- KPS ≥ 70
- Ability to comply with TTFields therapy
- Eligibility and indication for diagnostic or therapeutic neurosurgery and subsequent best practice oncological therapy
- tumor characteristics indicating significant expected benefit from feasible craniectomy or SR-surgery combined with TTFields therapy, i.e. (a) focal tumor and (b) most superficial border of tumor or resection cavity closer than 2 cm from the brain surface. The requirement for focal and superficially located disease is imposed to ensure an expected benefit from SR-surgery
- use of validated anti-conception for fertile female participants in concordance with guidelines provided by the Danish Health and Medicines Authority
- Signed written consent form

#### Exclusion criteria

- Pregnancy or nursing (fertile female participants will be required to take a validated pregnancy test for evaluation of pregnancy)
- Infra-tentorial tumor
- Implanted pacemaker, defibrillator, deep brain stimulator, other implanted electronic devices in the brain, or documented clinically significant arrhythmias
- Uncontrollable symptomatic epilepsy refractory to standard medication,
- Contraindications for skull remodeling surgery, e.g., bleeding diathesis or severe infection
- Significant co-morbidities, i.e. (a) significant liver function impairment (ALAT > 210 umol/L for men and > 135 umol/L for women or total bilirubin > 25 umol/L), (b) significant renal impairment (serum creatinine > 1.7 mg/dL = 150 umol/L), (c) coagulopathy (INR > 1.8 or APTT > 57 s), (d) thrombocytopenia (platelet count < 100 × 103/μL = 100 × 270,109/L), (e) neutropenia (ANC < 1.5 × 103/μL = 1.5 × 109/L), (f) anemia (Hb < 10 g/L = 6.0 mmol/l)
- Severe cognitive impairment
- Active participation in another therapeutic interventional clinical trial

### Surgery

All trial participants will receive a maximal safe resection or tumor biopsy. This serves a dual purpose a) to confirm histopathology recurrence of GBM per 2016 WHO criteria [16] and exclude non-eligible patients. b) to reduce trial participant risk by only performing SR surgery as an addition to biopsy or maximal safe resection.

**Fig. 1:**
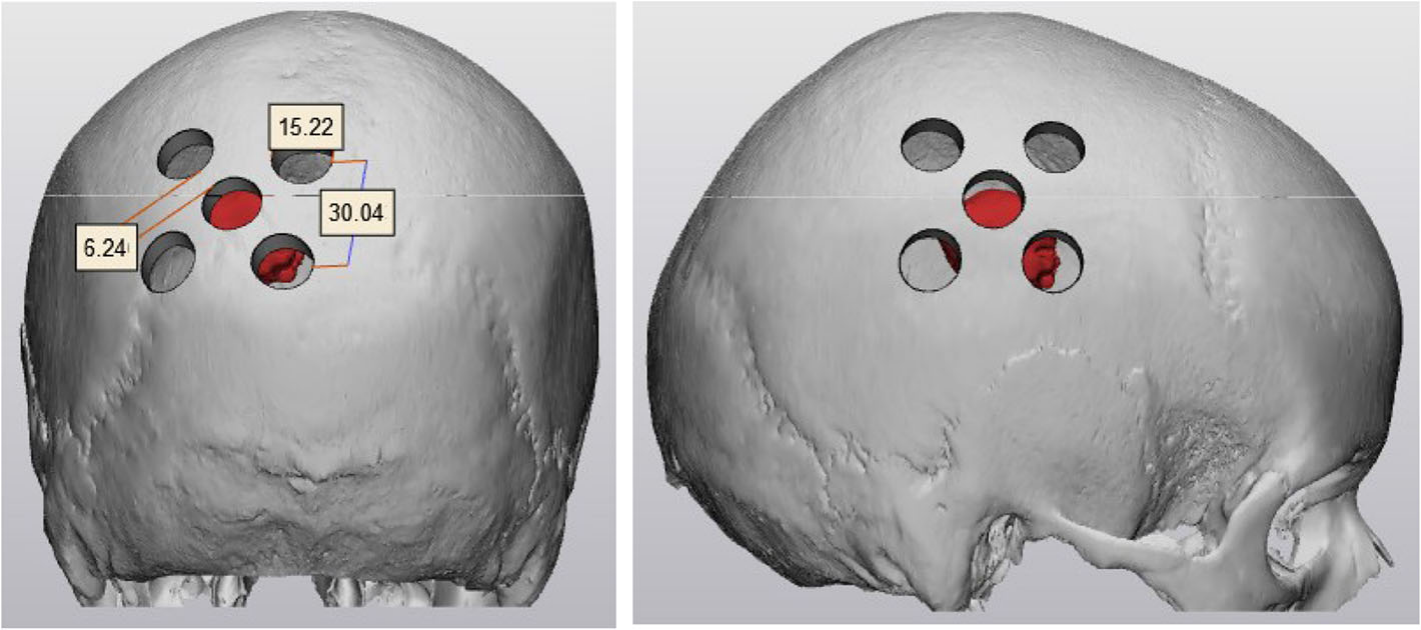
SR-surgery configuration shown. The central burrhole is placed directly over the tumor or tumor resection cavity. Each of the five burrholes is 15 mm in diameter

The SR surgery has been standardized and described in a trial standard operating procedure. It will involve five burrholes, each 15 mm in diameter. The configuration is shown in Fig. 1. The central burrholes will be placed directly over the tumor or tumor resection cavity. The configuration is based upon simulations using realistic patient head models or virtually introduced tumors in healthy head models and various skull remodelling configurations. These simulations showed that the configuration in Fig. 1 is optimal. The details of the work performed are beyond the scope of this article but will be described in subsequent published work.

Post-operative MRI (within 72 h) will be performed for all patients undergoing tumor resection to assess the extent of resection. If the extent of resection is unsatisfactory, a repeated surgical resection may be indicated if it can be performed safely [17]. Repeated surgery will thus not affect the patient’s eligibility for continued participation in the trial. A new post-operative MRI will be conducted preferably within 72 h after repeated resection. All neuroimaging used for response assessment will comply with the consensus recommendations for a standardized brain tumor imaging protocol in clinical trials [18]. A post-operative 3D CT scan of the cranium will also be performed to assess and visualize the skull remodelling results. Toxicity, complications to surgery, and estimation of KPS will be performed postoperatively.

### Randomization and blinding

Randomization (1:1 ratio) will be performed perioperatively after completion of resection or tumor biopsy to minimize that the allocation will influence the surgeon’s operation intentions. Randomiation is done via a Redcap module, which is set to permuted blocks of varying sizes (4/6/8) in random order across the entire trial. Each site will only have insight into their previous randomization making predictions difficult. The frequency of the various block sizes is unknown to all but the trial independent Aarhus University Redcap administrator. Once the randomization is done in Redcap it is impossible to edit. Patients will be randomized in a 1:1 ratio to receive TTFields and best practice medical oncological treatment with or without skull remodeling surgery. Local principal investigators, co-investigators, and sub-investigators will not be blinded, as optimal array planning will require knowledge about skull-remodelling surgery. The operating surgeon performing the skull-remodelling surgery will not be blinded. Patients will not be blinded, as it will be possible for the patient to feel the skull configuration and thereby determine whether SR-surgery was performed. Similarly, TTFields sham treatment is not possible due to different reasons like for example the heat emanating from the arrays.

### TTFields and medical oncological therapy

Patients with confirmed recurrence of GBM will proceed to receive TTFields and PBC. TTFields therapy and PBC will be initiated between 3 and 5 weeks after surgery. A baseline MRI of the brain and a baseline clinical assessment will be performed within 4 weeks before initiation of TTFields therapy. Often, the post-operative MRI will constitute the baseline MRI. The TTFields array layout will be planned per standard operating procedures to maximize the field intensity in the region of pathology [19–21]. We will use standard settings for the Tumor Treating Fields device, i.e. a frequency of 200 kHz and a peak-to-peak current of 1.8–2 A. TTFields will be combined with physician’s best choice medical oncological therapy, such as bevacizumab, temozolomide, irinotecan, lomustine, or combinations thereof, per the EANO guidelines [7, 22, 23].

### Follow-up and response assessment

During active TTFields treatment, patients will be followed regularly as per local guidelines and with clinical examinations, laboratory tests, and MRI, typically every 12 weeks or with shorter intervals. Deviations to the 12-week interval may be applied to accommodate logistical challenges or specific considerations for the individual case. Also, QoL will be assessed using QLQ-C30 and QLQ-BN20. Toxicity evaluation (CTCAEv5) will be conducted every twelve weeks. The follow-up regimen may be revised at the discretion of the treating physician. Particularly, additional follow-up including clinical and neuroradiological investigations, QoL assessments, toxicity evaluation, and neuroimaging may be done in connection with suspected or validated progression. Treatment response will be evaluated using the *modified criteria for radiographic response assessment in glioblastoma clinical trials*, which have been modified from the original RANO criteria to account for the potential delayed response previously observed for TTFields therapy [24, 25].

### Central review

MRI scans will undergo a central retrospective review by an independent trained neuroradiologist and performed in accordance to the recent consensus recommendation for brain tumor imaging [18]. If a discrepancy is observed between the local site and central review neuroradiologist, the images will be reviewed centrally by a third independent neuroradiologist. Decisions regarding the treatment and trial eligibility of participants will be made continuously by the local investigators, based on local response assessment. The central review will serve as *post-hoc* data quality assurance.

### Sample size and statistical considerations

The sample size calculations were based on a randomized, comparative, minimax two-stage design [14] and the binomial primary outcome of OS12 for the intent to treat trial participants *per* protocol, i.e. for whom TTFields therapy was initiated. This means that patients excluded prior to TTFields therapy are not included in the primary outcome analysis. Therefore, additional patients may be enrolled to ensure that the planned sample receives active TTFields treatment. The trial aims to indicate whether the intervention is superior to control and thus worthy of further phase 3 investigation. The expected OS12 in the control arm is set to 40% based on the EORTC 26101 trial [3], in which patients with first recurrence of GBM were treated with lomustine monotherapy or lomustine/bevacizumab combination therapy. The trial showed an OS12 of approximately 30%. We therefore set the expected level of OS12 to be slightly above this threshold given the fact that TTFields is added to the treatment, which otherwise represents recommended practice. No current studies provide accurate OS12 estimates for a population comparable to the study control arm. Therefore, we based the estimate on an expected benefit of 10% from TTFields alone compared to EORTC 26101. Setting the probabilities of false-positive and false-negative trial results to α = 0.15 and 1-β = 0.80, respectively, and defining the expected target level of OS12 to be OS12 = 0.6, i.e. a 316 20% absolute increase (and 50% relative increase) compared to the control arm, we calculated a maximum sample size of *n* = 42 patients in each arm (total *n* = 84) and an *expected* total sample size (en = 69.8). The expected sample size is calculated as en = n1 × PET0 + n × (1 − PET0), where n1 = 52 is the collective sample size of both groups at interim (stage 1) analysis, PET0 = 0.44 is the probability of early futility termination. The numbers are based on the given statistical parameters and a two-stage minimax design as given in SH Jung 2018 [26]. Interim futility analysis will be conducted after endpoint assessment of 52 patients and the trial will be terminated if the experimental arm performs worse than the control arm. In this case, the futility criterion will be defined as OS12experimental < OS12 control, and this conclusion will determine futility at marginal power and significance levels of 1-β* = 0.80 and of α* = 0.20, respectively. If the trial is not terminated at interim stage 1 analysis, the trial will proceed to enroll a total of 84 patients and terminate when the final OS12 endpoint data have been obtained for all patients. The secondary outcome measures of hazard rate ratios of PFS and OS will be tested using the log-rank test at the 0.05 alpha level. Time-to-event endpoints will be estimated from the time of inclusion. Toxicity and adverse events will be reported using absolute numbers and appropriate risk estimates. Final analysis will be conducted when all patients have been excluded or censored, e.g. due to end-of-trial or loss-to-follow-up. Subgroup analysis based on prognostic factors will be performed, incl. Analysis of patient characteristics in each arm and identification of characteristics of potential responders to the intervention. This will also include a correlation analysis between the calculated field distribution and outcome estimates.

### Withdrawal from study

The study will be terminated when all enrolled participants have been excluded from the trial and the necessary data have been acquired. A patient may be excluded from the trial due to one of the following reasons:

- Non-GBM diagnosis provided by tumor resection/biopsy
- Death,
- Loss to follow-up,
- End-of-trial,
- Enrollment in another interventional clinical trial,
- Withdrawal of consent
- The patient is no longer suitable for further participation due to ethical or medical safety reasons determined by the investigator.

### Data monitoring committee

An Independent Data Monitoring Committee (DMC) will be created with the main purpose of patient safety. The DMC will achieve this by monitoring ongoing data, especially regarding adverse events and analyzing the benefit vs risk ratio. Furthermore, they will provide an independent scientific review of the interim analysis and recommend continuation or discontinuation of the trial. If the trial passes interim analysis, the DMC will review the final data as well. The DMC will serve in an advisory capacity to the sponsor. Members of the committee will include two clinicians with neuro-oncological trial experience, a statistician, a nurse and two laypeople.

### Trial steering committee

The Trial Steering Committee (TSC) will consist of the sponsor-investigator and two representatives from each study site, in addition to one independent clinician outside of Denmark with neuro-oncological trial experience. The TSC will act in an advisory capacity to the sponsor in terms of reviewing the progress of the trial and, if necessary, recommend amendments to the protocol or trial logistics to ensure optimal trial progress. Furthermore, the recommendations provided by the DMC will be discussed and, if needed, implemented by the TSC.

### Publication

All results will be published in peer-reviewed international scientific journals and presented at international scientific conferences, regardless of academic conclusions. Positive, negative, and inconclusive results will be publicly available.

## Discussion

With no standardized treatment for recurrence glioblastoma and a poor prognosis, new treatment modalities or methods are highly needed. We present a randomized, interventional, clinical phase 2 trial testing a new and innovative intervention, skull remodeling surgery, to enhance TTFields therapy for first recurrence glioblastoma. Since the control arm uses a multimodal approach with TTFields therapy + BPC, we believe both trial arms are expected to show a survival benefit, regardless of randomization, compared to historically studies using either BPC or TTFields monotherapy. However, the interventional arm may benefit even greater if our phase 1 trial 15.5 months overall survival is any indication of the potential enhanced effect of SR-surgery.

All patients included would have undergone a tumor biopsy or resection, regardless of trial involvement. This reduces the risk of SR surgery to a minimum and strengthens the trial by a) reducing one statistical variable by not including patients where surgery is not indicated and b) ensuring all trial participants have a confirmed glioblastoma recurrence.

The limitations of the study are a very selected first recurrence GBM patient group, i.e. KPS ≥70, no significant comorbidity, surgery indicated, intact cognition, and no multifocality.

Another limitation is that the trial participants will not be stratified when randomized, which might give unbalanced baseline characteristics and if not adjusted give misleading results. However, with the interim analysis set at 52 patients and final data not to exceed 84 patients, there are too few trial participants to sensibly implement randomization based on MGMT status, degree of resection at primary and recurrence surgery, age, KPS and steroid usage at inclusion. There is a high risk that the two arms sample sizes will be imbalanced when trying to use block randomization with too many prognostic factors and too few trial participants. Ultimately, we decided not to stratify, which might give results that are incomparable. However, we are prepared to do retrospective stratification analysis if the baseline characteristics are unbalanced, in an attempt to gain meaningful data.

Finally, due to the nature of the surgery and how the TTFields device functions, blinding of the surgeons or patients to the intervention is not possible nor a trial design including a sham device or surgery.

## Data Availability

Not applicable.

## Abbreviations

KPS: Karnofsky performance score
OS12: Overall survival after 12 months
SUSAR: Suspected unexpected serious adverse reactions
SAE: Serious adverse events
GBM: Glioblastoma
OS: Overall survival
PFS: Progression-free survival
TTFields / TTF: Tumor Treating Fields
SR-surgery: Skull remodeling surgery
PBC: Physician’s best choice medical oncological therapy
QoL: Quality of life
KPS: Karnofsky Performance Score
ORR: Objective response rate
DMC: Independent Data Monitoring Committee
TSC: The Trial Steering Committee
GCP: Good Clinical Practice
